# Recurrent stroke risk in intracranial atherosclerotic disease

**DOI:** 10.3389/fneur.2022.1001609

**Published:** 2022-09-01

**Authors:** Ahmad A. Ballout, David S. Liebeskind

**Affiliations:** ^1^Department of Neurology, Northwell Health, and Donald and Barbara Zucker School of Medicine at Hofstra/Northwell, New York, NY, United States; ^2^Department of Neurology, University of California in Los Angeles, Los Angeles, CA, United States

**Keywords:** intracranial atherosclerosis (ICAS), hemodynamics, perfusion, intracranial atherosclerotic disease (ICAD), quantitative magnetic resonance angiography

## Abstract

Recurrent stroke risk secondary to intracranial atherosclerotic disease remains high despite aggressive medical treatment. This risk is further amplified in subgroups possessing biomarkers of hemodynamic insufficiency and potential for embolization, which have been shown to be independently and synergistically predictive of recurrent stroke. Luminal stenosis was predominantly used as entry criteria in major treatment trials, discounting the potential role of hemodynamics from primary analyses, limiting the strength of evidence and conclusions of these biomarkers to *post-hoc* analyses and other natural history studies. Future treatment trials should consider stratifying patients using a combination of these high-risk biomarkers. In the absence of trials, risk stratifying patients based on the presence of these markers may lend to more individualized clinical decisions. We aimed to summarize the studies that have investigated the relationship between biomarkers and their role in predicting recurrent stroke risk in intracranial atherosclerotic disease.

## Introduction

Intracranial atherosclerotic disease (ICAD) is the most common cause of ischemic stroke globally with the highest prevalence in Asians, Africans, and Hispanics (1, 2). The disease carries a poor natural history with high recurrent stroke rates in both medical and endovascular treated patients (3–5). The Comparison of Warfarin and Aspirin for Symptomatic Intracranial Arterial Stenosis (WASID) trial showed that warfarin was associated with significantly higher rates of intracranial hemorrhage and provided no benefit over aspirin in preventing ischemic strokes, with two-year cerebrovascular event rates over 20% in both arms (4). The results of this trial encouraged the exploration of endovascular treatments in this population at a time of rapid advancement in endovascular technologies. In a multicenter study of prospectively enrolled patients into the US Wingspan Registry, investigators reported periprocedural stroke rates as low as 5%, suggesting an acceptable rate of periprocedural morbidity in this population (6). These findings were challenged with the results of the SAMMPRIS and VISSIT trials which showed significantly higher rates of stroke with angioplasty and stenting compared to dual antiplatelet therapy, shaping national guidelines which endorse aggressive medical therapy and advise against endovascular treatment in this population (5, 7, 8). Furthermore, the more recent CASSISS trial showed no benefit of angioplasty and stenting compared to aggressive medical management, further validating current recommendations (9). None of these trials, however, risk-stratified patients based on hemodynamics or thromboembolic potential, relying exclusively on luminal stenosis as major entry criteria.

Although these studies targeted higher risk patients by including patients with at least moderate-to-high-grade stenosis, there is likely a high degree of heterogeneity within this group. For example, patients with a dominant hemodynamic mechanism may be at a greater risk of recurrent stroke compared to patients with thromboembolic mechanisms (10–12). Several prospective and retrospective studies have attempted to risk stratify these patients using various clinical and radiographic biomarkers, such as infarction patterns, hemodynamics, embolic signaling, plaque morphology, and vessel wall analysis (11, 13). Our aim was to review the current literature of the various biomarkers that have been used to stratify recurrent stroke risk. Future randomized studies of endovascular versus medical treatment of patients with ICAD may benefit by stratifying patients using these proven biomarkers.

## Methods

We searched the terms: “Intracranial atherosclerotic disease”; “ICAS”; ICAD”; “hemodynamics in intracranial atherosclerotic disease”; “risk stratification of intracranial atherosclerotic disease”; WASID; SAMMPRIS; MyRIAD; TOSS-2; “embolic signaling in intracranial atherosclerotic disease”; “hypoperfusion in intracranial atherosclerotic disease” in Pubmed.

## Defining symptomatic intracranial atherosclerotic disease

Ischemic events that localize to a downstream atherosclerotic intracranial large artery narrowed to at least 50% of its vessel diameter can be classified under the stroke mechanism of ICAD (4). Although the degree of stenosis may correlate loosely with hemodynamics and (14) has been used in landmark randomized controlled trials (4), defining a heterogenous disease based exclusively on luminal narrowing discounts other potentially risk-defining features, such as regional hemodynamics and plaque instability. Since the presence of collaterals and variability of vascular anatomy strongly influence upstream blood flow, the degree of focal stenosis may not correlate with regional hemodynamics (11). Several imaging modalities and methods have been used to measure hemodynamics, plaque stability, and potential for distal embolism, such as quantitative magnetic resonance angiography (QMRA) (11), CT and MR perfusion (15–18), standardized digital subtraction angiography (DSA) collateral scores (19), vasomotor reactivity and embolic detection using transcranial dopplers (TCDs), and high-resolution MRI. Prospective studies have used these methods to delineate the specific mechanisms of stroke and to identify high-risk patients (11, 13), but have largely been left out of treatment trials which relied exclusively on luminal size for inclusion (4, 5). The index events and primary outcomes used in treatment trials have also limited inclusion to transient ischemic attacks or clinical strokes, while other known sequelae of ICAD are disregarded such as subclinical infarction and cognitive function (20, 21). Indeed, this is a complex disease with a heterogenous patient population and should not be simplified to a strict definition based exclusively on lumen size.

### Degree of stenosis, infarction patterns, and hemodynamics

Ischemic strokes attributable to ICAD can occur due to various mechanisms, including hemodynamic ischemia, artery-to-artery embolism, branch atheromatous disease, and a combination of these mechanisms. There is likely a complementary relationship between these mechanisms, since hypoperfusion likely increases thrombosis and decrease the distal washout of thromboemboli (22–24). Infarction patterns are often used to infer stroke mechanism such that a borderzone pattern implies hemodynamic insufficiency, territorial pattern implies thromboembolism, and a perforator pattern implies branch atheromatous disease. The relationships between luminal stenosis, infarction patterns, hemodynamics, and recurrent stroke risk in intracranial atherosclerotic disease have been investigated in prospective studies and *post-hoc* analyses of the major trials below:

#### WASID *post-hoc* analyses

The first comprehensive study that systematically evaluated angiographic collaterals in patients with intracranial atherosclerotic disease was conducted by Liebeskind et. al in a WASID *post-hoc* analysis which found a strong relationship between the degree of stenosis and angiographic collaterals, and an inverse relationship between angiographic collaterals and anterograde flow across a stenosis (TIMI score) and into the downstream territory (TICI score) (25). This conceptualized angiographic collaterals as novel biomarkers in subclassifying patients with intracranial atherosclerotic disease. In a subsequent *post-hoc* analysis, the extent of collaterals was found to be independent predictors of recurrent stroke in a symptomatic arterial territory in the total population, however, two divergent patterns emerged depending on the degree of stenosis: collaterals were protective in preventing recurrent stroke in patients with severe stenosis but predicted an increased risk of recurrent stroke in patients with moderate stenosis (10). Although mechanisms that explain these apparent paradoxical findings remain uncertain and speculative, this study suggested that collaterals may identify more unstable milder stenoses. A subsequent study performed to evaluate the relationship between infarction pattern and collateral status did not find a statistically significant relationship between angiographic collaterals and baseline infarction patterns, including a relationship between borderzone infarctions and poor collaterals (26). Artery-to-artery embolism was thought to be the predominant stroke mechanism in this population, since 51% of the baseline infarctions and 62% of recurrent infarctions were in the territory of a single artery. In summary, these studies showed that angiographic collaterals correlate strongly with the degree of stenosis and are predictive of recurrent infarction but were unable to draw a relationship between infarction pattern and collateral status.

#### SAMMPRIS *post-hoc* analyses

In a SAMMPRIS *post-hoc* analysis of anterior circulation infarctions, patients with qualifying events attributable to internal and cortical borderzones were at significantly higher rates of recurrent infarction compared to non-borderzone infarctions at a median follow-up of 31 months (26.4 vs. 10.4%, *p* = 0.054) (12). Impaired DSA collaterals were significantly associated with recurrent infarction compared to complete collaterals (27 vs. 6%, *p* = 0.014) and were found in 70% of patients with borderzone infarctions. The presence of a borderzone pattern coupled with impaired collaterals had the highest rate of recurrent infarction at 37%. In addition, the rate of recurrent infarction continued to increase beyond 1 year in patients with either borderzone patterns or impaired collaterals while rates remained steady in patients with non-borderzone patterns or complete collaterals. Among patients with borderzone infarctions as the qualifying event, the primary endpoint was lower in the stenting (18%) vs. medically managed group (26%), and a Kaplan-Maier curve of primary endpoints using this subgroup favored endovascular treatment, although this was not statistically significant (*p*-value 0.30). This study confirmed the strong relationship between angiographic collaterals and recurrent stroke as previously seen in the WASID *post-hoc* analyses above (10), challenged the results of previous WASID *post-hoc* analysis by establishing a relationship between infarction pattern and angiographic collaterals (26), and most importantly, found that the coexistence of borderzone infarction with impaired collateral flow substantially increased the risk of recurrent stroke. These differences in findings of the relationship between collaterals and infarction patterns that were not found in WASID were thought to be due to the inclusion of moderate grade stenosis in WASID and differences in the grouping of collaterals despite use of the same grading system (12).

#### VERiTAS (prospective) and VERiTAS *post-hoc* analyses

The Vertebrobasilar Flow Evaluation and Risk of Transient Ischemic Attack and Stroke (VERiTAS) study was the first prospective study to evaluate the relationship between hemodynamics and recurrent stroke risk in patients with ICAD (11). Vertebrobasilar hemodynamics were measured using QMRA to dichotomize patients into low-flow and normal-flow groups based on prespecified algorithms that intrinsically accounted for collaterals by incorporating basilar and non-fetal posterior cerebral artery flow. Low distal flow status was associated with a three times higher rate of recurrent stroke compared to normal distal flow status (28% vs. 9%, *p* = 0.04) with a hazard ratio of 11.55 (95% CI, 1.88–71.00; *p* = 0.008) in a risk factor-adjusted multivariate analysis which was resistant to the effects of disease severity and location. A related study was performed by the study group that found a correlation between vessel-specific flow and the severity of stenosis, however, distal flow status, incorporating collateral capacity, was not predicted by the severity or location of disease (27). The VERiTAS studies supported hypoperfusion as a key mechanism of stroke in patients with posterior circulation ICAD, validated QMRA-defined distal-flow status as a possible biomarker of recurrent posterior circulation stroke and emphasized the importance of regional hemodynamics and collaterals in preventing stroke.

#### MyRIAD (prospective) and MyRIAD *post-hoc* analysis

MyRIAD was a prospective study that used various hemodynamic and plaque instability biomarkers to determine the mechanisms of recurrent ischemia in patients with ICAD (13). Anterograde flow was measured using QMRA, distal perfusion using perfusion MR (PWI), and vasomotor reactivity (VMR) and microemboli signals (MES) using transcranial dopplers. The primary outcome of ischemic stroke at 1 year was reached in 8.8% while secondary outcomes of TIA in 5.9%. Interestingly, 24.7% of patients were found to have subclinical infarctions in the territory of the symptomatic artery at 6–8 weeks follow-up. There was no significant association between abnormal imaging biomarkers and recurrent stroke, TIA, or new infarctions. Combining abnormal imaging biomarkers—such as QMRA low-flow and PWI delays—did not show clear synergistic effects in predicting recurrent infarction, although a trend toward significance was appreciated when comparing the presence of two biomarkers with one or fewer (33 vs. 17%, *p* = 0.07). A *post-hoc* analysis showed that the baseline number of diffusion-weighted imaging lesions (>1: 40.0%, 1: 26.9% vs. 0: 4.4%, *p* < 0.01) and borderzone infarction patterns were significantly associated with new or recurrent infarction (63.6 vs. 25.0%, *p* = 0.01), implying that hypoperfusion and artery-to-artery embolism likely contribute to early subclinical infarction (28). In summary, MyRIAD investigators were unable to prospectively identify a subgroup of ICAD at high-risk of recurrent ischemic stroke using various imaging biomarkers, however, baseline borderzone or multifocal infarction patterns were retrospectively found to be strong predictors of recurrent subclinical infarction.

#### TOSS-2 trial

The Trial of Cilostazol in Symptomatic Intracranial Arterial Stenosis (TOSS-2) failed to show a difference in recurrent stroke between patients treated with dual-antiplatelet therapy vs. monotherapy (29). A subanalysis evaluating patients that underwent baseline imaging and imaging at 7 months found a 12.5% rate of subclinical infarction and 3.7% rate of clinical recurrent stroke in the territory of the initial symptomatic intracranial artery (21). After classifying initial infarction patterns by location (subcortical vs. cortical vs. subcortico-cortical) and multiplicity (single vs. multiple), subcortico-cortical patterns and multiple lesions were found to be independent predictors of new ischemic lesions (OR, 3.01; 95% CI, 1.33–7.01; *p* = 0.03; OR, 2.81; 95% CI, 1.34–5.9; *p* = 0.006) and clinical recurrent stroke. Severe stenosis was associated with subcorti-cocortical pattern and multiple lesions on baseline imaging (p <0.001) but was not predictive of recurrent infarction.

#### Tissue perfusion studies

**Figure 1:**
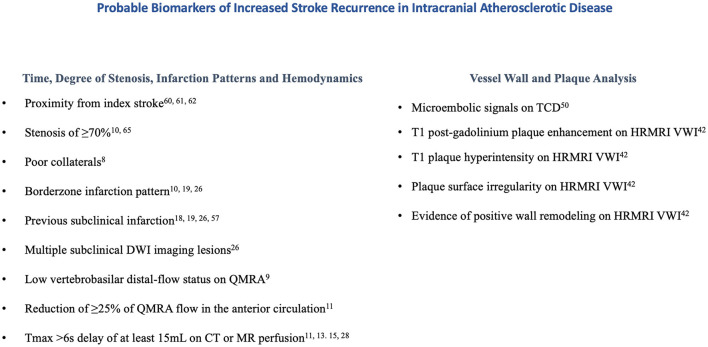
Probable biomarkers of increased stroke recurrence in intracranial atherosclerotic disease.

**Figure 2:**
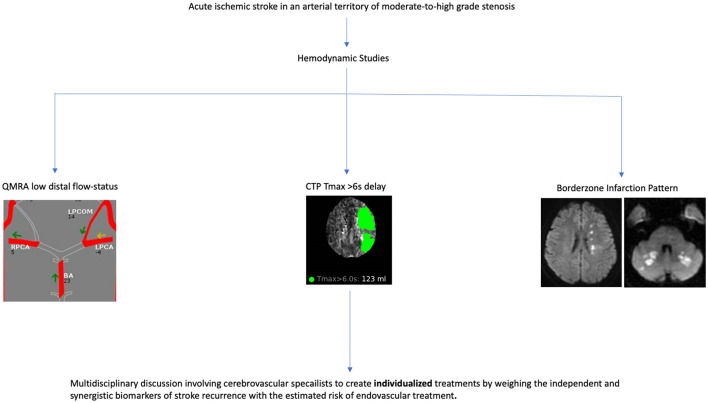
Workflow diagram.

Several retrospective studies have associated perfusion parameters and recurrent stroke risk in patients with intracranial atherosclerotic disease; Tmax >6s delays with mismatch volumes of at least 15 mL in the territory of a symptomatic intracranial artery were shown to be associated with recurrent cerebrovascular events (15, 17, 18) and increased lengths of hospital stay (30), while Tmax >4s delays were not (15, 30). Patients with anterior circulation internal borderzone infarction patterns were more likely to have recurrent cerebrovascular events and a target mismatch profile using Tmax >6s delays compared to non-internal borderzone infarctions (16), greater volumes of Tmax >4s and Tmax>6s delay compared to perforator patterns (31), and greater volume difference between Tmax >4s and Tmax>6s delay when compared to thromboembolic patterns (31). Although the MyRIAD study was unable to prospectively find an association between Tmax >6s delays and recurrent stroke risk, a trend toward significance was appreciated when Tmax >6s delays were coupled with QMRA low-flow states (13). The utility of perfusion imaging in predicting recurrent cerebrovascular events is less well established in the posterior circulation. In a prespecified sub-analysis of VERiTAS patients, rCBV and MTT ratios did not differ between posterior circulation QMRA low flow vs. normal flow states, inferring that MR perfusion may not be a reliable metric in evaluating regional hypoperfusion in the posterior circulation (32). Probable biomarkers of stroke recurrence are displayed in Figure 1 and a proposed workflow diagram is shown in Figure 2.

### Vessel wall integrity, plaque morphology, and plaque instability

High resolution vessel wall imaging by MRI is a novel non-invasive imaging tool that has been used to distinguish ICAD from alternate stroke etiologies and to stratify stroke risk (33, 34). Our pathophysiological understanding of the radiographic findings is based largely on extracranial carotid studies that collectively suggested that plaque enhancement likely occurs due to neovascularization, inflammation, and endothelial dysfunction resulting in gadolinium leakage, while T1 hyperintensity and surface irregularity presumably reflect intraplaque hemorrhage and rupture of the fibrous cap (35–39). Several meta-analyses have shown that intracranial plaque enhancement, positive remodeling, T1 hyperintensity, and surface irregularity are strong imaging biomarkers of symptomatic plaques in patients with ischemic events (40, 41) may be even more important than luminal changes in predicting stroke occurrence (42). In fact, intracranial plaque enhancement was associated with a four-times higher rate of recurrent stroke at 1 year compared to non-enhancing plaques in a prospective longitudinal study (43). Plaque enhancement has also been shown to correlate with multifocal infarction patterns perhaps implicating fragile plaques (44) and has been inversely correlated with time from stroke onset (45).

Microembolic signals (MES) detected by transcranial doppler were first used as markers of plaque instability in extracranial symptomatic and asymptomatic carotid stenosis as MES correlated with several biomarkers of plaque morphology, including plaque ulceration (46) and neovascularization (47) and were independently associated with an increased risk of stroke (48–50). In symptomatic intracranial atherosclerotic stenosis, MES occurs in over a quarter of patients (13, 51, 52), with increased frequency with degree of stenosis (51) and multifocal infarction patterns (53). In a prospective study of acute stroke patients with middle cerebral artery stenosis, MES were predictive of future cerebral ischemia (51), while the more recent MYRIAD study failed to show this association in a population of anterior and posterior circulation atherosclerotic stenosis (13). Combination therapy with clopidogrel and aspirin was more effective than aspirin alone in reducing MES in a randomized study of predominantly symptomatic intracranial atherosclerotic stenosis in the anterior circulation (52), in line with previous randomized studies of MES in extracranial carotid stenosis (54).

### Subclinical infarction and cognitive decline

Subclinical infarctions can occur due various stroke mechanisms (20) and have been associated with future stroke risk and cognitive decline (55–57). The primary endpoint of the major ICAD treatment trials predominantly focused on clinical strokes or TIAs, while subclinical infarction was often disregarded from primary analyses (4, 5). Recent evidence has shown that subclinical infarction occurs frequently in the early stages of an ischemic stroke and is predictive of future stroke (20, 21, 28, 58). In a SAMMPRIS sub-analysis, previous infarction more than doubled the risk of recurrent ischemic stroke (58). Subclinical infarction was found in a quarter of patients with ICAD at 6–8 weeks following an index stroke (28), and in more than half of patients in an Asian population (20), rates 3–5 times higher than that of recurrent clinical ischemic strokes (4, 5). Comparable to previous studies that have reported associations between infarction patterns and recurrent clinical stroke (12, 26), multiple DWI lesions (20, 21, 28, 59) and subcorticocortical infarction (21) patterns were shown to be independent predictors of subclinical infarction, implying similar hemodynamic and thromboembolic mechanisms. Interestingly, while antiplatelets have been shown to significantly reduce the rate of recurrent clinical stroke, neither antiplatelets nor anticoagulants influenced the occurrence of subclinical infarction in a retrospective study (20). Although subclinical infarction and white matter hyperintensities have been strongly associated with cognitive decline and future risk of neurocognitive disorders, most of these studies have focused on small vessel etiologies (60). Recent studies have shown this relationship in patients with asymptomatic extracranial large artery atherosclerosis (55–57), encouraging future studies in the intracranial vasculature. The inclusion of these radiographic and clinical biomarkers as entry or endpoint markers in future treatment trails may help capture a wider scope of pathology related to intracranial atherosclerotic disease.

### Time from index event

Ischemic strokes due to ICAD recur more frequently in the early period after the index stroke (61, 62), with drastically higher rates within 1 week of the event (63). Similarly, the risk of periprocedural stroke after intracranial stenting increases within this early time window, likely due to unstable plaques (hot plaques) and potential for embolization (5). ICAD treatment trials must be viewed in this context, since early enrollment may overestimate the risk of periprocedural complications and late enrollment may underestimate the risk in both endovascular and medically managed groups. For example, recurrent ischemic stroke risk was significantly higher in WASID patients that were randomized within the median enrollment time of 17 days compared to patients randomized after 17 days (62). Likewise, the periprocedural stroke risk in SAMMPRIS was more than five times that of WEAVE, with median enrollment times of 7 and 22 days from index event, respectively (5, 64). A SAMMPRIS analysis of periprocedural strokes, however, found no relationship between time from qualifying event and periprocedural ischemic stroke risk, and the benefit of medical therapy over endovascular treatment was similar in patients enrolled within 7 days of their qualifying event compared to patients enrolled beyond 7 days (65). The variability in other factors, such as operator experience (66), likely partially account for the large differences in periprocedural stroke risk among treatment trials, making it difficult to assert firm conclusions based exclusively on enrollment time alone. The recently published CASSISS trial limited enrollment of patients to beyond 3 weeks after their index event and found no difference in stroke recurrence between endovascular and medically treated patients. Both groups, however, had significantly lower rates of stroke recurrence compared to historical controls (5, 7), likely in part due to later enrollment (9). In summary, both the risk of recurrent ischemic stroke and periprocedural stroke appears to be highest in the early period after an index stroke, and therefore the risks of each must be weighed carefully in real-world treatment decisions and in the design of future prospective studies.

## Conclusions

Recurrent stroke risk in intracranial atherosclerotic disease remains high despite aggressive medical and endovascular therapies. Defining a vastly heterogenous disease exclusively based on luminal size discounts the role of other high-risk biomarkers, such as hemodynamics and potential for embolization, limiting the generalizability of current treatment trials. Incorporating these biomarkers in isolation as entry criteria into future trials may pose a challenge given the uncertain thresholds of hemodynamic insufficiency and thromboembolic potential. Perhaps the integration of biomarkers, given the probable synergism, may better target a hemodynamically insufficient subgroup most resistant to antithrombotic therapies, justifying endovascular flow augmentation, despite its current risks. While randomized controlled trials should remain the gold standard in guiding treatment, biomarkers of stroke recurrence may perhaps be used as an adjunct in clinical decision-making to better estimate recurrent stroke risk, allowing for more targeted and individualized treatment. The myriad of stroke mechanisms in intracranial atherosclerotic disease may be too complex and multidimensional to be managed by simplified and universal treatment strategies.

## Author contributions

AB and DL contributed to conceptualization, literature search, writing—original draft, and writing—review and editing. Both authors contributed to the article and approved the submitted version.

## Conflict of interest

The authors declare that the research was conducted in the absence of any commercial or financial relationships that could be construed as a potential conflict of interest.

## Publisher's note

All claims expressed in this article are solely those of the authors and do not necessarily represent those of their affiliated organizations, or those of the publisher, the editors and the reviewers. Any product that may be evaluated in this article, or claim that may be made by its manufacturer, is not guaranteed or endorsed by the publisher.
